# Differential Expression of Mucin in Salivary Gland Tumours

**DOI:** 10.3390/medicina60060920

**Published:** 2024-05-31

**Authors:** Nurul Inaas Mahamad Apandi, Siew Wui Chan, Yen Fa Toh

**Affiliations:** 1 Fakulti Pergigian, Universiti Kebangsaan Malaysia, Kuala Lumpur 50300, Malaysia; 2 Fakulti Pergigian, Universiti Malaya, Kuala Lumpur 50603, Malaysia; swuichan@um.edu.my; 3 Jabatan Patologi, Pusat Perubatan Universiti Malaya, Kuala Lumpur 50603, Malaysia; yenfa@um.edu.my

**Keywords:** pleomorphic adenoma, mucoepidermoid carcinoma, Alcian blue, sialomucin, sulfomucin

## Abstract

*Background and Objectives*: Mucin has been implicated via various mechanisms in the development and growth of tumour cells. However, mucin expression studies in salivary gland tumours are limited, especially with samples from minor salivary glands. This study aims to investigate and compare mucin expression in benign and malignant salivary gland tumours of minor and major salivary gland origins. *Materials and Methods*: Special stains were used to stain neutral mucin (Periodic acid Schiff), sialomucin (Alcian Blue) and sulfomucin (Aldehyde Fuschin) within tissues from six normal salivary glands and 73 salivary gland tumours including 31 pleomorphic adenomas, 27 mucoepidermoid carcinomas, and 15 adenoid cystic carcinomas. A semi-quantitative approach was used to evaluate mucin expression within ductal lumens. Sialomucin was the most expressed mucin in all salivary gland tumours, regardless of origin. *Results*: A significant difference was observed in the mucin expression between benign and malignant salivary gland tumours, as pleomorphic adenoma showed three times significantly higher expression of sialomucin compared to mucoepidermoid carcinoma and adenoid cystic carcinoma (*p* = 0.028). Pleomorphic adenomas of major glands showed 42 times significantly higher expression of sialomucin compared to those of minor glands (*p* = 0.000). *Conclusions*: Sialomucin content in pleomorphic adenomas of major glands was vastly increased compared to that in minor glands. Differential sialomucin expression in benign and malignant salivary gland tumours suggests a role in diagnosing of borderline salivary gland tumours.

## 1. Introduction

Salivary gland neoplasms are relatively infrequent, accounting for 2–6.5% of head and neck tumours, with approximately 75% benign [1]. Secretory mucin has been postulated to play a significant role in disease development [2,3], as mucin differs in quality and quantity under normal and neoplastic conditions [3]. Mucin can be differentiated into epithelial mucin and connective tissue mucin. Epithelial cell components secrete neutral and acidic mucins, with acidic mucin further classified into sialomucin and sulfomucin. On the other hand, connective tissue mucin contains more acidic mucosubstances including, chondroitin sulphates, keratin sulphates, hyaluronic acid and dermatan sulphates [3].

Development and growth of tumour cells have been linked to the dysregulation of mucin protein core expression. Through this selection process, enzyme-modified tumour cells are presumed to express novel mucin forms, enhancing their survival. Mucin may influence the biological properties of tumour cells in several ways. Unlike epithelial cells, mucin could confer protection and serve as a barrier to tumour cells. This allows the tumour cells to be sheltered from toxic compounds, rendering them resistant towards acids, chemotherapeutic agents and cytotoxic compounds [4].

The mucin layer could capture biologically active molecules such as growth factors and cytokines that may promote tumour growth, offering an indirect influence over the regulation and interaction of the immune system, inflammatory response and stromal cell interaction with tumour cells. Differentiation and proliferation of tumour cells are governed by cell surface mucin via morphogenetic signal transduction and ligand-receptor interaction. Overexpression of mucin leads to signalling interaction via the mitogen-activated protein kinase (MAPK) pathway [5], which can be triggered in response to external growth factors. Mucin has also been associated with tumour metastasis by its involvement in a molecular process that regulates anti-adhesion and adhesion effects [6]. The modulation of leukocyte activity is possible, as high levels of soluble mucin form a barrier that nullifies the effects of leucocytes on tumour cells. The presence of these mucins within the blood and lymphatics would also obstruct the antigen-presenting mechanism against tumour cells [4].

**Figure 1:**
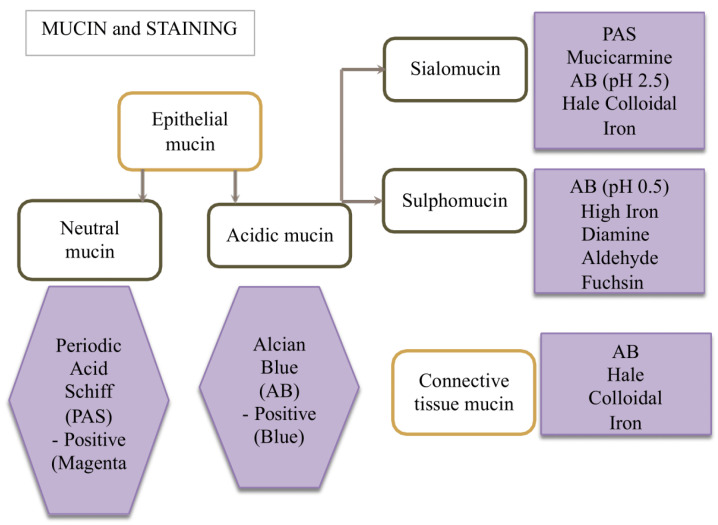
Diagrammatic representation of mucins and their corresponding stains, modified from various sources.

Mucin can be stained with different single stains and a combination of stains, thus enabling them to be classified. Both epithelial and connective tissue mucins can be stained for identification. Stains available for mucin include Periodic Acid Shiff (PAS), Alcian blue (AB), Mucicarmine (MC), High Iron Diamine (HID), Aldehyde Fuchsin (AF), Hale Colloidal Iron and Toluidine blue staining. Epithelial neutral mucin can be stained with PAS, while epithelial acidic mucin can be stained with PAS, MC and AB. For the distinction between the two types of acidic mucin, sialomucin can be stained with PAS, MC, AB (pH 2.5) and hale colloidal iron, while sulfomucin can be stained with AB (pH 0.5), AF and HID. Connective tissue mucin can be highlighted with AB and Hale colloidal iron [7]. A summary is shown in Figure 1. Hence, in this study, we will use a combination of PAS, AB and AF stains to achieve better contrast. A PAS/AB stain will be used to identify neutral and acidic mucin, while AB/AF will be used for identification of acidic mucin. AB at pH 2.5 is specific to highlight the presence of sialomucin, while AF is specific for traces of sulfomucin.

In salivary gland tumours, it has been shown that sialomucin was predominant within benign tumours and in combination with sulfomucin in the malignant types. This is shown in PA, whereby, there is a predominance of sialomucin and neutral mucin [3]. Meanwhile, higher traces of sialomucin and sulfomucin were found in MEC of salivary glands compared to MEC of the oesophagus [8]. AdCC also showed more sialomucin presence and neutral mucin in focal areas [9]. Another study demonstrated that periodic acid Schiff diastase (PAS-D) positive granules were helped differentiate various salivary gland neoplasms [10].

Mucin expression studies in salivary gland tumours are few and far between, with existing studies limited by sample size or restricted to a few types of salivary gland tumours due to the generally rare incidence of these tumours. No study explores salivary gland origin as a possible confounding factor for mucin expressions within the same type of salivary gland tumours. This study investigated mucin expression in benign and malignant salivary gland tumours of minor and major salivary gland origins to explore the potential of mucin histochemistry as an indicator of clinical behaviour in salivary gland tumours.

## 2. Material and Methods

### 2.1. Tissue Sample

Sample size calculation was done using G*Power software, version 3.1.9.4 for this study. Based on the effect size of 0.25 and power of 0.86, the estimated sample size was 32 for each group. Unfortunately, 5 PA cases, 9 MEC cases and 7 AdCC cases from minor salivary glands had to be replaced because the initial selected blocks were eaten by rats. After staining, 1 MEC case from a minor salivary gland had to be discarded due to insufficient tumour tissue. Two more cases (1 PA and 1 AdCC) from the major salivary gland were rejected as the wrong tissue blocks were provided. At the final count, the sample collection was at 79. The sample consisted of 6 control group tissues, from normal salivary gland tissues, of which, 3 were from submandibular glands (major salivary gland), 3 were from normal labial mucosa region (minor salivary gland) and 73 were histologically diagnosed salivary gland neoplasms which included 31 cases of pleomorphic adenoma (PA), 27 cases of mucoepidermoid carcinoma (MEC) and 15 cases of adenoid cystic carcinoma (AdCC) from 1995 to 2019. PAs consisted of 15 cases from major glands and 16 from minor glands. For MEC, 12 were from major glands and gradings are as follows; 6 low grade, 2 intermediate grade and 4 high grade, while 15 were from minor glands and all were low grade except 1, which was intermediate grade. For AdCC, 3 were from major glands while 12 were from minor glands, and all of cribriform patterns except those for 1 minor gland displayed a solid arrangement, as shown in Table 1. Major glands utilized for the majority of the samples were from the Parotid gland and Submandibular glands, while minor glands utilized the majority were from the palatal and retromolar region. The corresponding formalin-fixed paraffin-embedded tissue blocks were sourced from the archives of the Oral Pathology Research and Diagnostic Laboratory, Department of Oral and Maxillofacial Clinical Science, Faculty of Dentistry, University of Malaya (UM) and Anatomical Pathology Division of Department of Pathology, University Malaya Medical Centre (UMMC). Control group tissues composed of normal salivary tissue from intraoral mucoceles and normal submandibular glands obtained from surgical specimens with neck dissections. This study was approved by the Medical Ethics Committee, Faculty of Dentistry, UM [DF OS1911/0044(P)] and the Medical Research Ethics Committee, UMMC (20200622-8793).

### 2.2. Histochemistry

Histochemical re-staining was done on 4–5 μm-thick sections cut by a microtome and mounted on glass slides. Histological staining was performed with Harris Haematoxylin and Eosin. Harris Haematoxylin and Eosin were used as they provide crisp clear nuclear detail. The combination of Alcian Blue-Periodic Acid Schiff (AB-PAS) staining was performed according to Mowry [11] while a combination of Alcian Blue-Aldehyde Fuschin (AB-AF) staining was performed according to Spicer and Mayer [12]. PAS-stained magenta for neutral mucin, AB-stained blue for sialomucin (acidic mucin) and AF-stained purple for sulfomucin (acidic mucin). Staining reagents or kits were procured from Premier Diagnostics Sdn Bhd, Malaysia (Alcian blue (pH 2.5) 100 test/kit, Hamburg, Germany; Gomori’s paraldehyde fuschins, 1 kit (100 tests); Hematoxylin) and Labchem Sdn Bhd, Malaysia (Sigma Aldrich Schiffs Fuschin, sulfite reagent, Bayswater, Australia) suitable for detection of glycoproteins, 500 mL; Periodic acid for analysis, Merck, Rahway, NJ, USA).

### 2.3. Semi-Quantitative Analysis

The localizations of AB, PAS, and AF were examined under a virtual microscope (Olympus 534630, Hamburg, Germany) and the images were captured by using a digital microscope (Olympus BX51, Hamburg, Germany) at a high resolution of 4080 × 3072 pixels. Tumour tissue from biopsied specimens was entirely utilized while tissue from surgical specimens, a few different sections, were evaluated before selecting a final suitable representative section. Distribution of the stains within the tumour microenvironment was assessed, with the findings recorded as (+) indicating the presence of stain and (−) indicating the absence of stain. A semi-quantitative approach was used to further evaluate the expression of mucin within the ductal lumens. This approach was considered due to the categorical data nature of the percentage of surface areas highlighted by the special stains. For each sample, the entire surface area of the tumour microenvironment was examined under 20× to 100× magnifications using Toupview software (Version: V3.4_20230223) to assess the percentage of stains within lumens of ductal structures. Intra-examiner and inter-examiner calibrations involving three examiners were conducted and verified to minimise discrepancies. To avoid the possibility of inaccurate scoring, areas of intense inflammation and necrosis were excluded. The extent of scoring was categorized as follows: 0, less than 25% of surface area positivity; 1, 25–50% of surface area positivity; 2, 50–75% of surface area positivity and 3, more than 75% of surface area positivity. The scoring was further classified into two as follows: less than 50% of surface area positivity and more than 50% of surface area positivity.

### 2.4. Statistical Analysis

Result analysis was performed using IBM Statistical Package for Social Sciences (SPSS) version 26 obtained from SPSS Inc., Chicago, IL, USA. Comparative analysis was performed using Chi-square test and Fisher’s exact where applicable, for mucin expression (AB, PAS and AF) in relation to minor or major salivary gland tumours and benign or malignant salivary gland tumours. Post-hoc analysis was done using Binary logistic regression. For all the statistical analyses, *p* Value < 0.05 indicates statistical significance.

## 3. Results

### 3.1. Sample Details

The salivary gland tumours involved patients ranging from 15 to 81 years of age. Benign tumours like PA occurred significantly more often in younger patients while malignant tumours often affected the elderly. Details on the presentations of salivary gland tumours are summarized in Table 2.

### 3.2. Mucin Expression in Salivary Gland Tumours

**Figure 2:**
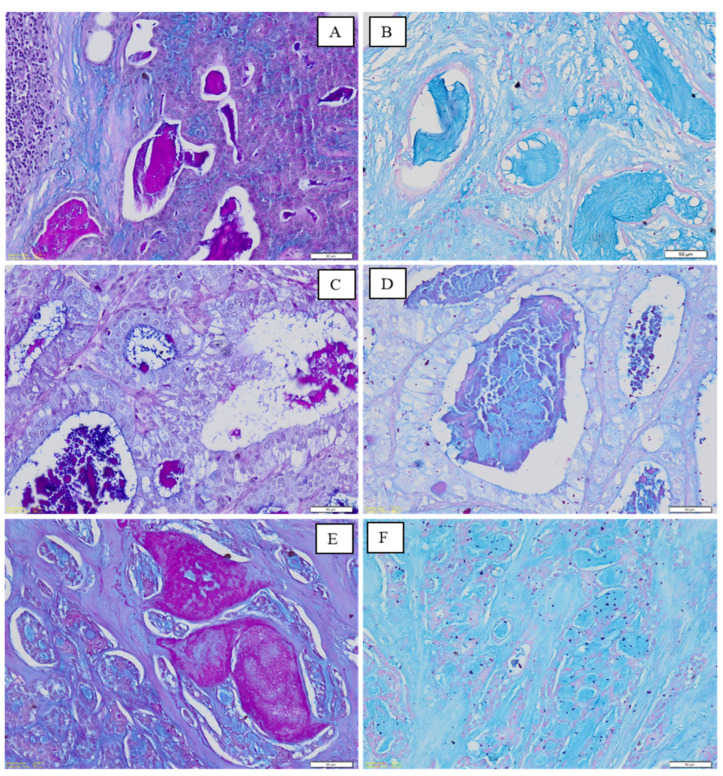
Photomicrograph shows staining in samples from minor salivary gland tumours: PA (**A**,**B**), MEC (**C**,**D**) and AdCC (**E**,**F**). (**A**) Luminal areas showing PAS-positivity with AB-PAS staining. (**B**) Predominance of AB staining within the lumens with AB-AF staining. (**C**) AB and PAS presence within lumen with AB-PAS staining. (**D**) Dominance of AB with traces of AF within lumens with AB-AF staining. (**E**) Dominance of AB with traces of PAS within the pseudocystic spaces with AB-PAS staining. (**F**) Dominance of AB within the pseudocystic spaces with AB-AF staining. [Magnification ×200].

**Figure 3:**
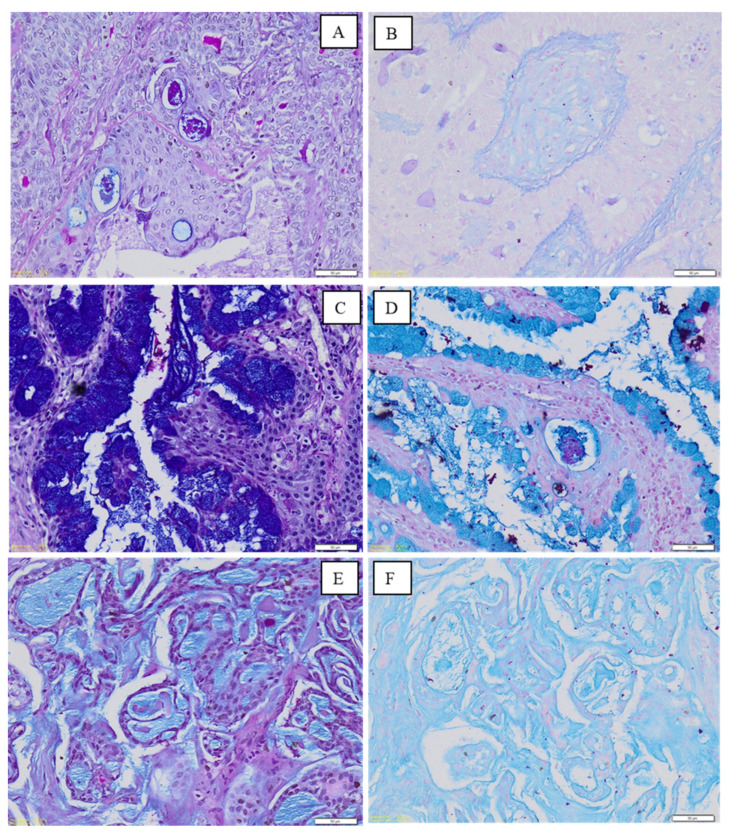
Photomicrograph shows staining in samples from major salivary gland tumours; PA (**A**,**B**), MEC (**C**,**D**) and AdCC (**E**,**F**). (**A**) Presence of both AB and PAS within luminal areas with AB-PAS staining. (**B**) Presence of AB and AF within luminal areas with AB-AF staining. (**C**) Mucous cells show AB-positivity with traces of luminal PAS-positivity with AB-PAS staining. (**D**) Dominance of AB and traces of AF within luminal areas with AB-AF staining (**E**) Dominance of AB within pseudocystic spaces with AB-PAS staining. (**F**) Dominance of AB within pseudocystic spaces in AB-AF staining. [Magnification ×200].

Variable amounts of all three types of mucins were present in PA, MEC and AdCC (Figure 2 and Figure 3). Generally, all the salivary gland tumours, regardless of origin, showed greater dominance of AB-positive mucin (sialomucin). A significant difference between the expression of AB within PA of minor and major salivary glands was also shown (Table 3). Expression of AB within PA of major salivary glands was 42 times higher compared to PA of minor glands.

Benign salivary gland tumours demonstrated three times more expression of AB compared to malignant salivary gland tumours (Table 4).

## 4. Discussion

Mucin is a complex molecule rich in protein and polysaccharide components, generally divided into epithelial and connective tissue mucin [3]. However, this study focused on neutral and acidic mucins secreted by epithelial cell components, considering only PA is a mixed tumour. Neutral mucins comprise hexosamines and hexose units without a free acidic group but with increased amounts of uncharged monosaccharides. Meanwhile, acidic mucin is further divided into sialomucin or sulfomucin based on the presence of terminal sialic acid or sulphate groups on the oligosaccharide chain respectively [13].

Salivary gland secretions are dependent on the composition and types of acini; serous, mucous or mixed. Generally, parotid glands are composed of mainly serous acini, sublingual glands of mainly mucous acini, and submandibular glands and minor glands of mixed acini [2,14]. A descriptive study found that mucin content in mucous cells was composed of sulfomucin and sialomucins, while serous acini contained other highly sulfated substances that were AB-positive but may not have been mucins [3]. The authors also noted that mucous acini in submandibular glands demonstrated varied heterogeneity; similarly, this is reflected in our findings [3]. In another study, neutral mucin was predominant, with little acidic mucin within parotid glands [13]. These staining variations reiterate the complexity and heterogeneous nature of salivary glands and their tumours, though it seems that mucous acini tend to produce acidic mucin, especially sialomucin.

Salivary gland tumours were shown to be sialomucin-dominant within benign tumours and demonstrated a sialomucin–sulfomucin combination within malignant tumours [3], but all our samples showed greater sialomucin expression, regardless of origin (Table 5). A possible explanation for the ubiquitous sialomucin content could be derived from the role of human mucin gene, MUC4, also known as a sialomucin complex, which shares similar ascitic sialoglycoprotein subunit and receptor domains. MUC4 expression was elevated throughout the epithelial thickness in normal or dysplastic mucosa close to the invasive tumour, indicating a cellular protective function of the mucin in reaction to the tumour and its predisposing environmental factors [15]. Sialomucin or its associated cells may possess similar protective functions.

The exceptionally high sialomucin content in major PA compared to minor PA cannot be attributed to the original acini composition of the glands, since minor glands arguably contain more mucous acini. We could only speculate that maybe PA in major glands consisted of abundant luminal structures in comparison to minor glands. Previously, Naag et al. (2010) found elevated neutral and sialomucin content within PA of major salivary glands [3]. The sialomucin and neutral mucin combination in MEC of either origin also differs from other studies that reported a mixture of sialomucin and sulfomucin in MEC [3]. Relatively higher sialomucin content was a consistent finding but sulfomucin was barely detected in MEC in this study. The disparate sulfomucin detection patterns could be due to the use of different staining kits. In contrast to our findings, Lam et al. (1993) noted that oesophageal MEC had higher sialomucin content compared to MEC of the salivary gland, which had higher sulfomucin and sialomucin content, and histogenetic origin of the mucin-producing components was suggested as a possible explanation [8]. Comparing findings of AdCC from either origin was not feasible due to the imbalanced cases but the elevated sialomucin content within pseudocystic spaces in AdCC coincided with previous studies [16]. Other studies involving AdCC reported dominant sialomucin with focal areas of faint neutral mucin [3,8] or the presence of both sulfomucin and sialomucin [17]. To the best of our knowledge, no similar research was done specifically on minor salivary glands. Comparing mucin expression between minor and major salivary glands would ensure that mucin profiling done on salivary gland tumours is not undermined by their origin. However, we acknowledge that our findings would probably apply best to salivary glands of similar variants, as cystic or tubular variants theoretically express more mucin.

Greater sialomucin expression in benign salivary gland tumours compared to the malignant ones suggests a possible role of sialomucin or sialomucin-producing cells in the malignant progression of salivary gland neoplasms, correlating well with many of the reported roles of mucin in growth and development of tumour cells. Mucin is known for its wide-ranging influence over tumour cells via control of the local environment, regulation of differentiation and cell proliferation, tumour suppression, invasion, metastases and regulation of inflammation and immune response [2]. This finding is also consistent with the reported observations in many tumours that acidic mucin content was elevated as tumorigenesis progressed [18,19].

Mucin expression has also been linked to the histological tumour grade and prognosis of MEC [20]. MUC1 was expressed in high-grade MEC and associated with poor prognosis and shorter disease-free survival intervals, while MUC4 was highly expressed in low-grade MEC with a better prognosis and longer disease-free survivals. Our findings suggest that AB staining may be useful in distinguishing benign salivary gland tumours from malignant ones, like the use of AB (pH 2.5) in differentiating benign prostate hyperplasia and well-differentiated prostate adenocarcinoma [21]. Mucin studies in salivary gland tumours were found to be extremely lacking for further comparisons as most researchers tend to gravitate towards more precise immunohistochemical studies. Existing mucin studies mainly concentrated on gastrointestinal tumours, where grading is based on the postulated theory that there would be sulfomucin predominance in malignancy and increased neutral mucin expression with progressive differentiation of gastrointestinal tumours [22,23,24]. In addition to neutral mucin promotion, sulfomucin demotion during progressive tumour differentiation was also reported in later studies [18,19]. Although insignificant, a greater extent of neutral mucin content among MEC was seen compared to PA in our study. Unfortunately, the concept of differentiation is not applicable to these tumours. Then again, the results were also varied and inconsistent even amongst gastrointestinal mucin studies. Gad (1982) reported that well-differentiated carcinomas expressed more sialomucin compared to sulfomucin [19], while another study showed a marked increase of sialomucin content in more extensive tumours [22].

Salivary mucin secretion may also be affected by underlying obstructive disease, which is commonly seen in submandibular glands [25]. Submandibular duct papillae are often involved due to the ductal course; that is, against gravity, in addition to its long and curved anatomical structure [26]. Although, in our samples, there was no history of obstructive disorder; however, it can be evaluated further, especially for the two samples of PA from the submandibular gland in our study.

We acknowledge that this study had some limitations. Firstly, although the sample size was larger than it was in previous studies, there was an unequal distribution within each type of salivary gland tumour, especially for AdCC. Secondly, the varied histological features of each type of salivary gland tumour could influence the amount of mucinous content highlighted by the special stains. This problem was particularly prominent when a minute or an absence of mucinous composition was noted within solid tumour variants. This study could be improved by selecting salivary gland tumours of the same variants. We also had issues with the Aldehyde Fuschin kit stain because the reagents given as counterstains for nuclei were red while AF was violet and these colours were of similar intensity, thus making interpretation a laborious procedure. Lastly, histochemistry is only limited to portraying the presence of mucosubstances and does not allow the depiction of the refined element of these mucosubstances. Therefore, future studies need to be carried out to further explore the complex nature of salivary gland tumours.

## 5. Conclusions

In conclusion, sialomucin was the most expressed mucin in all salivary gland tumours irrespective of origin, inferring that alcian blue is the best special stain to visualize mucin components during microscopic examination. Significantly higher sialomucin expression in benign salivary gland tumours compared to malignant tumours suggests a possible role in the diagnosis of borderline salivary gland tumours. Perhaps further insight could be derived by investigating the role of sialomucin or sialomucin-producing cells in the malignant evolution of salivary gland tumours, with the use of PA and carcinoma ex-PA samples. Sialomucin expression within PA of major glands was excessively elevated in comparison to minor glands, while mucin expressions in MEC and AdCC were not significantly affected by their origin. Hence, caution is advised in samplings of PA for future research of a similar nature.

## Figures and Tables

**Table 1:** Distribution of cases included in study.

Salivary Gland Tumour	Minor Glands	Major Glands
PA	16	15
MEC	15 (14 low-grade, 1 intermediate-grade)	12 (6 low-grade, 2 intermediate-grade and 4 high-grade)
AdCC	12 (All tubular and cribriform pattern)	3 (2 tubular and cribriform pattern, 1 solid pattern)

PA: Pleomorphic adenoma; MEC: Mucoepidermoid carcinoma; AdCC: Adenoid cystic carcinoma.

**Table 2:** Clinical presentations of salivary gland tumours in this study.

		PA (*n* = 31)		MEC (*n* = 27)		AdCC (*n* = 15)	
		Minor	Major	Minor	Major	Minor	Major
Age * *n*(%)	<47	14 (45)	9 (29)	7 (26)	2 (7)	2 (13)	1 (7)
	>47	2 (6)	6 (19)	8 (30)	10 (37)	10 (67)	2 (13)
Gender *n*(%)	F	12 (39)	11 (35)	4 (15)	11 (41)	8 (53)	1 (7)
	M	4 (13)	4 (13)	4 (15)	8 (30)	4 (27)	2 (13)
Ethnicity *n*(%)	Chinese	9 (29)	8 (26)	7 (26)	6 (22)	6 (40)	2 (13)
	Malay	4 (13)	4 (13)	6 (22)	5 (19)	6 (40)	1 (7)
	Indian	3 (10)	3 (10)	2 (7)	1 (4)	-	-
Intraoral sites *n*(%)	Palate	11 (35)	-	7 (26)	-	2 (13)	-
	Non-palate	5 (16)	-	8 (30)	-	10 (67)	-
Extraoral sites *n*(%)	Parotid	-	9 (29)	-	11 (41)	-	2 (13)
	Non-parotid	-	6 (19)	-	1 (4)	-	1 (7)

* Median age of 47 years was used as cut-off point for age binary measurement. PA: Pleomorphic adenoma; MEC: Mucoepidermoid carcinoma; AdCC: Adenoid cystic carcinoma.

**Table 3:** Comparison of mucin expression between minor and major salivary gland tumours.

Stain	AB, *n*(%)		*p* Value	AF, *n*(%)		*p* Value	PAS, *n*(%)		*p* Value
	Low	High		Low	High		Low	High	
PA									
Minor *n* = 16	13 (81)	3 (19)	^c^* 0.000	8 (53)	7 (47)	^c^ 0.484	9 (75)	3 (25)	^f^ 0.242
Major *n* = 15	2 (13)	13 (87)		8 (67)	4 (33)		1 (33)	2 (67)	
MEC									
Minor *n* = 15	15 (94)	1 (6)	^f^ 1.000	15 (100)	-	^α^ -	12 (100)	-	^α^ -
Major *n* = 12	15 (100)	-		12 (100)	-		3 (100)	-	
AdCC									
Minor *n* = 12	15 (94)	1 (6)	^f^ 1.000	12 (80)	3 (20)	^c^ 0.756	12 (100)	-	^α^ -
Major *n* = 3	15 (100)	-		9 (75)	3 (25)		3 (100)	-	

Significant level *p* = 0.05, ^c^: Pearson Chi-square; ^f^, Fisher’s Exact test; low, <50%; high, ≥50; ^α^ *p*-values were not computed as figures did not meet statistical test requirements; ^c^* Binary logistic regression showed higher AB expression within major PA compared to minor PA by 42, folds (*p* = 0.000); PA, Pleomorphic adenoma; MEC, Mucoepidermoid carcinoma; AdCC, Adenoid cystic, carcinoma; AB, Alcian blue; AF, Aldehyde Fuschin; PAS, Periodic acid–Schiff.

**Table 4:** Comparison of mucin expression between benign and malignant salivary gland tumours.

Stain	Benign *n* = 31		Malignant *n* = 42		*p* Value ^c^
	Low	High	Low	High	
AB, *n*(%)	12 (39)	19 (61)	29 (69)	13 (31)	0.016 *
AF, *n*(%)	31 (100)	-	41 (98)	1 (2)	0.387
PAS, *n*(%)	28 (90)	3 (10)	38 (90)	4 (10)	0.982

Significant level: *p* = 0.05, ^c^: Pearson Chi-square test, Low: <50%, High: ≥50%, Benign—PA, Malignant—MEC & AdCC, * Binary logistic regression showed higher expressions of AB within benign tumours than malignant, tumours by 3 folds (*p* = 0.028); PA: Pleomorphic adenoma; MEC: Mucoepidermoid carcinoma; AdCC: Adenoid cystic carcinoma; AB: Alcian blue; AF: Aldehyde Fuschin; PAS: Periodic Acid-Schiff.

**Table 5:** Summary of high mucin expression (>50%) in luminal spaces.

	Minor Glands	Major Glands
Normal	>Sialomucin (100%)	* Mixed neutral and acidic mucin (100%)
PA	>Sialomucin (19%)	>Sialomucin (86%)
MEC	>Sialomucin (47%) and neutral mucin (20%)	>Sialomucin (33%) and neutral mucin (25%)
AdCC	>Sialomucin (34%)	>Sialomucin (67%)

* Submandibular gland; PA, pleomorphic adenoma; MEC, mucoepidermoid carcinoma; AdCC, adenoid cystic carcinoma.

## Data Availability

The original contributions presented in the study are included in the article, further inquiries can be directed to the corresponding author.
